# *Agrobacterium*-Mediated Genetic Transformation of Embryogenic Callus in a *Liriodendron* Hybrid (*L. Chinense* × *L. Tulipifera*)

**DOI:** 10.3389/fpls.2022.802128

**Published:** 2022-03-17

**Authors:** Meiping Li, Dan Wang, Xiaofei Long, Zhaodong Hao, Ye Lu, Yanwei Zhou, Ye Peng, Tielong Cheng, Jisen Shi, Jinhui Chen

**Affiliations:** ^1^Key Laboratory of Forest Genetics & Biotechnology of Ministry of Education of China, Co-Innovation Center for Sustainable Forestry in Southern China, Nanjing Forestry University, Nanjing, China; ^2^College of Biology and the Environment, Nanjing Forestry University, Nanjing, China

**Keywords:** suspension culture, somatic embryogenesis, agrobacterium tumefaciens, GUS, liriodendron hybrid

## Abstract

A highly efficient genetic transformation system of *Liriodendron* hybrid embryogenic calli through *Agrobacterium*-mediated genetic transformation was established and optimized. The *Agrobacterium tumefaciens* strain EHA105, harboring the plasmid pBI121, which contained the *ß*-glucuronidase (*GUS*) gene and neomycin phosphotransferase II (*npt* II) gene under the control of the CaMV35S promoter, was used for transformation. Embryogenic calli were used as the starting explant to study several factors affecting the *Agrobacterium*-mediated genetic transformation of the *Liriodendron* hybrid, including the effects of various media, selection by different Geneticin (G418) concentrations, pre-culture period, *Agrobacterium* optical density, infection duration, co-cultivation period, and delayed selection. Transformed embryogenic calli were obtained through selection on medium containing 90 mg L^−1^ G418. Plant regeneration was achieved and selected *via* somatic embryogenesis on medium containing 15 mg L^−1^ G418. The optimal conditions included a pre-culture time of 2 days, a co-culture time of 3 days, an optimal infection time of 10 min, and a delayed selection time of 7 days. These conditions, combined with an OD_600_ value of 0.6, remarkably enhanced the transformation rate. The results of *GUS* chemical tissue staining, polymerase chain reaction (PCR), and southern blot analysis demonstrated that the *GUS* gene was successfully expressed and integrated into the *Liriodendron* hybrid genome. A transformation efficiency of 60.7% was achieved for the regenerated callus clumps. Transgenic plantlets were obtained in 5 months, and the PCR analysis showed that 97.5% of plants from the tested G418-resistant lines were PCR positive. The study of the *Liriodendron* hybrid reported here will facilitate the insertion of functional genes into the *Liriodendron* hybrid *via Agrobacterium*-mediated transformation.

## Introduction

*Liriodendron*, belonging to the magnolia family, consists of two species of large deciduous trees that produce hardwoods of high commercial and ecological value. *Liriodendron chinense* is native to eastern Asia, and is documented in the List of National Key Protected Wild Plants in China (http://www.forestry.gov.cn/, Announcement No. 15, 2021). *Liriodendron tulipifera*, or yellow poplar, known as the tulip tree due to its beautiful flowers, is widely distributed in eastern North America (Chen et al., 2019a). Large quantities of *L. tulipifera* wood are used for furniture, pulping, plywood, and construction lumber (Dai et al., 2004). *Liriodendron* is considered a basal angiosperm, occupying a crucial position for studying the evolutionary history of flowering plants (Zhou et al., 2016).

A new hybrid strain of *Liriodendron* was successfully cultivated by Ye et al. in 1963 through crossing between *L. chinense* and *L. tulipifera*. This hybrid is a rapidly growing hardwood tree, making it desirable for timber plantations and an attractive potential source of biomass for energy (Merkle et al., 1993; Li et al., 2012). Owing to its advantages in environmental adaptability and pest and disease resistance, this *Liriodendron* hybrid also has ornamental and industrial value (Hao et al., 2020). In addition, it has potential medicinal value as an ethno-health-promoting plant (Yang et al., 2015). Great advances in forestry genetics, breeding, and plantation and landscape application have been made in *Liriodendron* (Wang, 2003).

Seasonal restrictions, a low natural seed-setting rate, and a low germination rate greatly limit the expansion of *Liriodendron* plantations (Chen et al., 2019b). However, for most plants, an *in vitro* regeneration system is the primary means of propagation (Thiruvengadam et al., 2013; Zhou et al., 2014). In particular, somatic embryogenesis, which plays a critical role in the study of plant embryology and is considered an effective method of vegetative propagation, has been used in *Liriodendron* (Merkle and Sommer, 1986; Chen et al., 2003a; Dai et al., 2004). Culture methods have gradually improved since the *Liriodendron* hybrid was first successfully regenerated in 1993 using immature zygotic embryos as explants *via* somatic embryogenesis (Merkle et al., 1993). A suspension culture system of embryogenic calli of the *Liriodendron* hybrid was established with high proliferation efficiency (Chen et al., 2007b; Li et al., 2007). Using this method, under artificially controlled culture conditions, mass somatic embryos can be successfully produced. This system provides an efficient tool for the study of the molecular, morphological, and physiological regulation of the regeneration pathway during the onset and development of embryogenesis in *Liriodendron*.

An efficient transformation protocol is a prerequisite for acquiring transgenic plants and the genetic improvement of many species. A stable genetic transformation method is convenient for identifying the functional roles of candidate genes in somatic embryogenesis (Ratjens et al., 2018). Compared with microprojectile bombardment and electroporation, *Agrobacterium*-mediated transformation is a common tool for plant transformation with many advantages, such as easy operation, low cost, low-copy transgene integration, the transfer of large DNA fragments (Xia et al., 2020), and insertion priority into transcriptionally active regions (Cha et al., 2012). *Agrobacterium*-mediated transformation has been widely used in research and applied to many woody species to acquire transgenic plants (Shou et al., 2004; Prasad et al., 2014), such as *Populus* (Fillatti et al., 1987), *Eucalyptus globulus* (Matsunaga et al., 2012), *Quercus robur* (Vidal et al., 2010), elm (Bolyard et al., 1991), Chinese chestnut (*Castanea mollissima*; Sun et al., 2020), and European larch (Shin et al., 1994).

In recent years, there has been some progress on the genetic transformation system in *Liriodendron*. However, compared with other species, the transgenic method in *Liriodendron* is the least developed. Previous studies showed that transformation based on microprojectile bombardment was successfully performed with *L. tulipifera* calli and protoplasts, from which transgenic plants were obtained, albeit at a low transformation efficiency of 0.004% (Wilde et al., 1991, 1992; Rugh et al., 1998). *Agrobacterium*-mediated transformation in *Liriodendron* hybrid callus has been reported, but it is still unstable (Chen et al., 2007a). A polyethylene glycol-mediated transient transfection system in the protoplasts also has been established (Huo et al., 2017). However, these systems all have the same shortcomings originating from the relatively low transformation efficiency. The establishment of a transgenic system will improve the functional genomic research and molecular breeding of *Liriodendron* hybrids. Therefore, further optimization of the protocol for a genetic transformation system for *Liriodendron* is critical and imperative, and would provide an essential step forward and a reference for future research in the Magnoliaceae and other woody trees.

Many factors can influence the efficiency of plant transformation. These include *Agrobacterium* strains and plasmids, cell density, co-culture conditions and inoculation, plant species and genotype, explants, and selection agents (Cheng et al., 2003; Karami, 2008; Bett et al., 2019). In order to establish a successful approach for the genetic transformation of the *Liriodendron* hybrid, an appropriate regeneration system *in vitro* is an important prerequisite (Vidal et al., 2010). Furthermore, a successful selective culture strategy for identifying resistant transformants is another key factor for genetic transformation (Wu et al., 2021). We found that different developmental stages during the somatic embryogenesis of the *Liriodendron* hybrid exhibited different tolerances to the same type of selective agent. Thus, developing a novel selection strategy to improve the transformation efficiency of the *Liriodendron* hybrid based on this characteristic would be a key advance. When attempting to establish a new transformation protocol in any species, it is necessary to optimize the parameters influencing the efficiency of genetic transformation, as this can reduce future material and labor costs (Karami, 2008). In addition, successful transformation requires a balance among the factors affecting the transformation frequency (Shrawat et al., 2007).

In this study, some important parameters influencing the *Agrobacterium*-mediated genetic transformation of the *Liriodendron* hybrid were studied using embryonic callus as the starting explant. An efficient *Agrobacterium* transformation system was established that could provide a basic technique for gene function analyses. This approach could also be used for genome editing and for transferring new traits into a wide range of hybrid *Liriodendron* genotypes.

## Materials and Methods

### Plant Materials and Culture Conditions

In this research, the controlled artificial pollination of the female parent (*L. chinense*) with pollen from the selected male parent (*L. tulipifera*) was conducted in a breeding orchard of Nanjing Forestry University in late April 2012. Immature aggregated samaras were collected from the pollinated tree 8 weeks after pollination. Then, these samaras were surface-sterilized and dissected on a clean bench. The immature zygotic embryos with endosperm were immediately excised and transferred to the callus induction medium (CIM). The embryogenic callus was initiated from immature embryos of the *Liriodendron* hybrid 1 month after the subculture. Finally, the calli of a somatic line (genotype No. 52053) were selected as the starting explant in this study.

The plantlets obtained from the embryogenic calli were based on the established somatic embryogenesis system of the *Liriodendron* hybrid (Chen et al., 2003a). The embryogenic callus was maintained on CIM. After the induced calli were sub-cultured onto the same type of fresh CIM medium for 2 weeks, the calli were transferred to liquid CIM medium and suspension-cultured at 23°C in the dark with shaking at 95 rpm for 2 weeks. The suspension calli were sub-cultured in fresh liquid CIM medium every week. After 2 weeks of incubation, the cultures were sieved successively through stainless-steel sieves of 100- and 200-μm pore size, and suspended cells were collected in a sterile Erlenmeyer flask using a Buchner funnel. Suspended cells (2 ml by volume) were collected on 9-cm sterile filter paper using micro pipette tips, transferred to embryo induction medium (EIM), and incubated at 23°C in the dark. After 3–4 weeks of incubation, plantlets were obtained and then transferred to shoot elongation medium (SEM), following which they were sub-cultured under a 16-h/8-h (light/dark) photoperiod at 23°C before being transferred to soil pots in the greenhouse.

The embryogenic callus was sub-cultured at 23 ± 1°C in the dark for 2 weeks and then used for genetic transformation, as well as for testing explant sensitivity to G418. The pH of the medium was adjusted to 5.7 prior to autoclaving at 121°C at 0.11 MPa pressure for 20 min. All media used in this study are shown in Supplementary Tables 1 and 2.

### *Agrobacterium* Strains and Vectors

**Figure 1:**
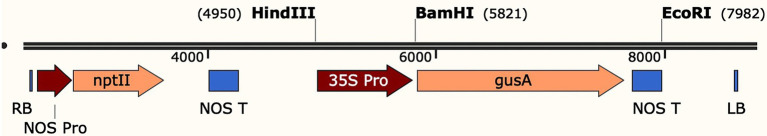
Schematic diagram of the T-DNA region in the plasmid pBI121-*GUS*. RB, right border; LB, left border; NOS-Pro, promoter sequences of the nopaline synthase gene; *nptII*: neomycin phosphotransferase II gene; NOS-T: nopaline synthase terminator; HindIII = unique HindIII restriction site within T-DNA; BamHI = unique BamHI restriction site within T-DNA; EcoRI = unique EcoRI restriction site within T-DNA; 35S-Pro, cauliflower mosaic virus 35S promoter; *gus A*, coding region of the *GUS* gene.

The *A. tumefaciens* strain EHA105 harbored the plasmid pBI121 (14,758 bp; Figure 1) and contained *β-glucuronidase* (*GUS*) as the reporter gene. The *Cauliflower mosaic virus* (CaMV) 35S promoter, as well as the *neomycin phosphotransferase II* (*nptII*) gene under the control of a nopaline synthase (NOS) promoter (Chen et al., 2003b) and terminator, were used for initial optimization (Jefferson, 1987b). The plasmid vector was transferred into *A. tumefaciens* strain EHA105 using the heat shock method (Wei, 2009). Bacteria were cultured on an agar-solidified Luria-Bertani (LB) medium (Hooykaas et al., 1977), supplemented with 50 mg L^−1^ kanamycin and 20 mg L^−1^ rifampicin (Sigma, United States) at 28°C in the dark.

### G418 Sensitivity Test

To identify the optimum concentration of the selective agent for the transgenic callus, the sensitivity of the embryogenic callus to G418 was tested. The embryogenic callus clumps were cultured on CIM supplemented with different concentrations of G418. Cefotaxime (400 mg L^−1^) was used to inhibit *Agrobacterium* after cocultivation with the callus (data not shown). Embryonic calli (0.55 g) were incubated in CIM medium containing 200 mg L^−1^ cefotaxime and different levels of G418 at 0, 30, 60, 90, and 120 mg L^−1^. Subculture was performed with the same fresh medium every 4 weeks, embryogenic calli were examined, and the relative increases in the weights of fresh embryogenic callus clumps were recorded to analyze the callus proliferation rate after 8 weeks of culture. Each petri dish contained 10 embryonic callus clumps. Three petri dishes were used for each treatment on embryogenic calli, and three independent replications were performed for each treatment.

To determine the sensitivity of the somatic embryos to G418, 2 ml of suspended cells (0.3 × 10^5^ cells/ml) was inoculated on EIM supplemented with 0, 5, 10, 15, or 20 mg L^−1^ G418. The numbers of somatic embryos were examined to calculate the optimum concentration of G418 after 6 weeks of culture. Ten petri dishes were used for each treatment, and all experiments were repeated with three independent replicates. All antibiotics were filter-sterilized (0.22 μm) and added to the autoclaved medium after the medium had been cooled to 45°C before solidification.

### Evaluation of Parameters Influencing Transformation

To establish an optimum *Agrobacterium*-mediated transformation protocol for the *Liriodendron* hybrid, the following factors, which influenced the transformation frequency, were evaluated: the time that the calli were pre-cultured on co-culture medium (CCM; 0, 2, 4, or 6 days); the optical density of the *A. tumefaciens* cell culture (OD_600_ values of 0.2, 0.4, 0.6, 0.8, or 1.0); the duration of infection (0, 5, 10, 15, 20, 25, or 30 min); the duration of co-cultivation (1, 2, 3, or 4 days); and the duration of delayed selection (0, 7, 14, 21, or 28 days). The transient *GUS* expression was used to optimize the parameters that affected the transformation frequency. At the end of the delayed selection stage, the resistant callus regeneration rate was recorded to calculate the delayed selection time. Transient *GUS* activity was determined in *A. tumefaciens*-inoculated explants after cocultivation with *Agrobacterium.* Each variant of each parameter was optimized by screening for transient *GUS* expression and tested using three independent replicates.

### Transformation Protocol

#### Preparation of the *A. tumefaciens* Culture

The *A. tumefaciens* strain EHA105 harboring the plasmid pBI121 was used for transformation. Positive colonies of the individual strain were cultured in liquid LB medium (40 ml) supplemented with 50 mg L^−1^ kanamycin and 20 mg L^−1^ rifampicin on a rotary shaker at 28°C and 180 rpm in the dark overnight. One milliliter of bacterial suspension was grown in 50 ml of the same medium to an optical density at 600 nm (OD_600_) of 0.6–1.0 under the same culture conditions. The bacterial suspension was centrifuged immediately at 5000 rpm for 10 min, and the pellet was resuspended in ¾ strength Murashige and Skoog (MS) liquid medium (Supplementary Table 1; Murashige and Skoog, 1962) containing 100 μmol L^−1^ acetosyringone (AS) to a final OD_600_ = 0.6. The bacterial suspension was poured into a sterile Erlenmeyer flask and used for staining.

#### Transformation of Embryogenic Callus

For genetic transformation, embryogenic calli pre-cultured on CCM for 2 days were infected with *A. tumefaciens* strain EHA105, and the starting explants were immersed in a solution of cultured cells grown to an OD_600_ of 0.6. These calli were submerged in the bacterial suspension and shaken for 10 min, then immediately blotted dry using sterilized filter paper to remove excess bacterial suspension. They were then transferred to CCM as mentioned above. After the calli had been co-cultivated with the *Agrobacterium* strain on CCM at 23°C in the dark for 2 days, the calli were rinsed four times with sterile water containing 400 mg L^−1^ cefotaxime and then blotted dry on sterilized filter paper to decontaminate the *Agrobacterium*. Then, the calli were transferred to delayed selection medium (DSM) and cultured for 7 days at 23°C in the dark. After 7 days of delayed selection, the calli were incubated on callus selection medium (CSM) to induce the transgenic calli, and the transgenic calli were then sub-cultured once over a 25-day interval.

During this procedure, all factors were optimized using optimization assays. In each assay, one parameter was changed, while the other parameters remained constant based on the findings of the preliminary test. As such, the only variable in each assay was the parameter to be optimized.

#### Plant Regeneration of Transgenic Somatic Embryos

After 8 weeks of incubation, the resistant calli were transferred to liquid CIM medium supplemented with cefotaxime (200 mg L^−1^) and suspension-cultured at 23°C in the dark. Two weeks later, the suspended calli were transferred to EIM (see the section “Plant materials and culture conditions”) supplemented with cefotaxime (200 mg L^−1^) and G418 (15 mg L^−1^) at 23°C in the dark. After 3–4 weeks, most of the somatic embryos had germinated and were then transferred to the light and then to the SEM, following which they were cultured at 23°C under a 16-h light/8-h dark photoperiod until the plantlets could be transferred to greenhouse conditions. The plating suspension cell density was maintained at 0.6 × 10^5^ cells per plate, and 10 replicates were performed for each treatment, and incubated at 23°C in dark.

### Histochemical Analysis of Putative Transformants

For histochemical analysis of *GUS* gene expression, the GUS enzyme activity was detected in the transformed callus and plants using standard and modified methods (Jefferson, 1987a; Matsunaga et al., 2012). Transformation efficiency was assessed by *GUS* signal intensity on a hemocytometer grid in three random replicates with 10 μl of suspension cells at 200× magnification under a light microscope (Zeiss Axio Vert. A1). Cells stained *gus* blue were recorded as positive. The calli and plants were then stained using 5-bromo-4-chloro-3-indolyl glucuronide-*β*-glucuronidase (X-gluc) at 37°C for 12 h, and the non-transformed calli and plantlets were used as negative controls. The calli and plant tissues were bleached, fixed in 70–95% ethanol, and then examined under a microscope (Leica M165FC).

### Molecular Analysis of Transgenic Plants

Genomic DNA was extracted from 100 mg of embryonic calli and fresh leaves of putative transformants and non-transformed (control) plantlets using a modified cetyltrimethylammonium bromide (CTAB) method (Pamidimarri et al., 2009). Thirty clumps of transgenic calli and 40 pieces young leaves of putative transgenic plantlets were screened by PCR to detected the presence of *GUS* and *nptII* genes in transgenic calli and plants.

The PCR reactions contained 0.5 μl of dNTPs (10 mM), 12.5 μl 2× Phanta Max Buffer, 1 μl of each primer (10 μM), 0.5 μl Phanta Max Super-Fidelity DNA Polymerase (1 U/μl, Vazyme, Nanjing, China), and 20–50 ng of genomic DNA in a 25-μL volume. A 740-bp region was amplified using the specific primers of the *nptII* gene (*NPTII*-F: 5′-AGAGGCTATTCGGCTATGACTG-3′ and *NPTII*-R: 5′-GAACTCGTCAAGAAGGCGATAG-A-3′). A pair of *GUS* gene primers (*GUS*-F: 5′-ATCTCTATGAACTGTGCGTCACAG-3′ and *GUS*-R: 5′-CTTCTCTGCCGTTTCCAAATCG-3′s) was used to amplify a 707-bp DNA fragment from the *GUS* gene. The PCR conditions were as follows: −95°C for 3 min, 35 cycles of 95°C for 15 s, 58°C for 15 s, 72°C for 1 min, and a final extension at 72°C for 7 min (one cycle). The PCR-amplified products were separated by electrophoresis on 1.2% agarose gels at 180 V for 20 min.

The RNA was isolated from the fresh leaves of the transgenic and control plants using a FastPure® Plant Total RNA Isolation Kit (Vazyme, Nanjing, China) and was quantified spectrophotometrically (NanoDrop, Thermo Scientific, Wilmington, DE, United States). The cDNA was synthesized using a HiScript® III 1st Strand cDNA Synthesis Kit (+gDNA wiper; Vazyme Nanjing, China).

For reverse transcriptase–polymerase chain reaction (RT–PCR) analyses, the obtained cDNA was amplified using the primers 5′-AAACGGCAGAGAAGGTACTGG-3′ and 5′-TCTTCACTCCACATGTCGGTG-3′, which generated a fragment of the *GUS* gene (136 bp). The primers 5′-ATTCCAGAGGACCAGTTCCTG-3′ and 5′-AGCAAGTGAGAGATTGTCCTTG-3′, which amplified the UBQ gene (234 bp), were used as an internal control. The total volume of the reaction mixture was 20 μl and included Takara Taq™ (10× PCR Buffer (Mg^2+^ free), 2 μl MgCl_2_ 30 mM, 1.5 μl of dNTP Mixture (2.5 mM), 1 μl primers (10 pmol), cDNA (100 ng), 0.1 μl Taq DNA polymerase (#R001AM, TAKARA BIO INC.), and RNase-free water (added to a total volume of 20 μl). The PCR program was as follows: 95°C for 5 min, 27 cycles at 95°C for 30 s, 60°C for 30 s, 72°C for 25 s, and a final extension at 72°C for 10 min. The PCR products were separated on 1.2% agarose gel electrophoresis in 1xTAE buffer.

For quantitative real-time PCR (qRT-PCR) analyses, the obtained cDNA was amplified using the GUS gene primers (F: 5′-AAACGGCAGAGAAGGTACTGG-3′, R: 5′-TCTTCACTCCACATGTCGGTG-3′) and the UBQ gene (F: 5′-ATTCCAGAGGACCAGTTCCTG-3′, R: 5′-AGCAAGTGAGAGATTGTCCTTG-3) as an internal control. The amplification was performed using a LightCycler® 480 Real-Time PCR System (Applied Biosystems), with the data set to comparative cycle threshold Ct (ΔΔCt). The qRT-PCR reaction mixtures were carried out in a 20-μL volume containing 2 × AceQ qPCR SYBR Green Master Mix (without ROX; 10 μl; Vazyme Nanjing, China), 10 ng of cDNA, and primers (4 pmol). The qRT-PCR conditions were as follows: 95°C for 5 min, and then 40 cycles at 95°C for 10 s, and 60°C for 30 s. The non-transformed plantlet was used as a control sample, the *UBQ* gene was selected as a calibrator sample for ΔΔCt analysis, and the relative quantification value was calculated and exported for further analysis. Three technical replicates were performed for each plant and gene.

#### Southern Blot Analysis

Genomic DNA was extracted from different transgenic plants and non-transformed (control) plantlets using a DNAsecure Plant Kit (TIANGEN BIOTECH, Beijing, China), and approximately 10 μg of genomic DNA was digested with HindIII. The plasmid DNA was used as a positive control, and genomic DNA from the non-transformed (control) plantlet was used as a negative control. Digoxygenin (DIG)-labeled probes were obtained using PCR DIG Probe Synthesis Kit (Roche, Mannheim, Germany), through PCR amplification of a genomic fragment using the primers 5′-ACGGCAAAGTGTGGGTCAA-3′ and 5′-AGCGTAAGGGTAATGCGAGG-3′, which generated a fragment of the PCR-labeled probes (722 bp). The digested DNA fragments were separated in a 0.7% agarose gel and transferred to a nylon membrane (Hybond-N+; Amersham, United Kingdom; Fang et al., 2019). Southern hybridization was digoxigenin-labeled using a DIG-High Prime DNA Labeling and Detection Starter Kit II (Roche, Mannheim, Germany).

### Data Analysis

All data for the optimization experiments were analyzed using one-way analysis of variance (ANOVA), Graph Pad Prism 8.0 (Graph Pad Software, Inc., United States), and Microsoft Excel. Tukey test was used for statistical analysis among multiple treatments. A *p*-value of <0.05 was regarded as statistical significance.

Callus relative fresh weight is the difference between secondary callus fresh weight and initial callus fresh weight. This value could be estimated according to the formula: Relative fresh weight (g) = Fresh weight of embryogenic callus after 8 weeks culture (g) – initial callus fresh weight (g). The *Agrobacterium* transformation efficiency was estimated according to the following formulas: The rate of transient transformation frequency (%) = (the number of *GUS* signal positive transgenic callus cells/total number of callus cells for staining) × 100%; the rate of plant transformation efficiency (%) = (the number of the PCR-positive transformants/total number of putative transformants) × 100%.

## Results

### Sensitivity of Embryogenic Callus and Somatic Embryo to G418

**Figure 2:**
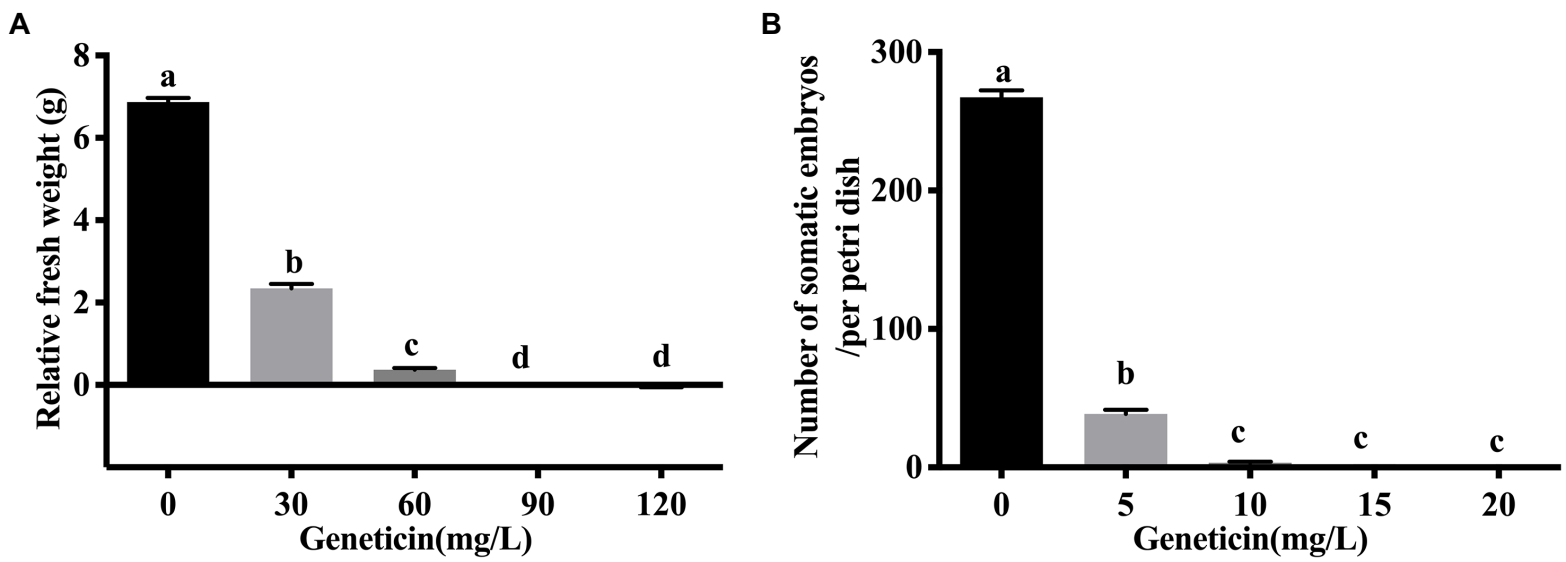
The sensitivity of embryogenic calli and somatic embryos of the *Liriodendron* hybrid to Geneticin (G418). **(A)** The relative fresh weight of the embryogenic callus under the different concentrations of G418. **(B)** The regenerative capacity of somatic embryos of the *Liriodendron* hybrid under G418 stress at 0, 5, 10, 15, and 20 mg L^−1^. Callus relative fresh weight is the difference between secondary callus fresh weight and initial callus fresh weight. Each replicate includes 30 embryogenic callus masses per dish. Data correspond to the mean ± SD of three replicates. An ANOVA test was used for statistical analysis. Column bars with the different letters indicate significant differences at *p* < 0.05 by Tukey test.

**Figure 3:**
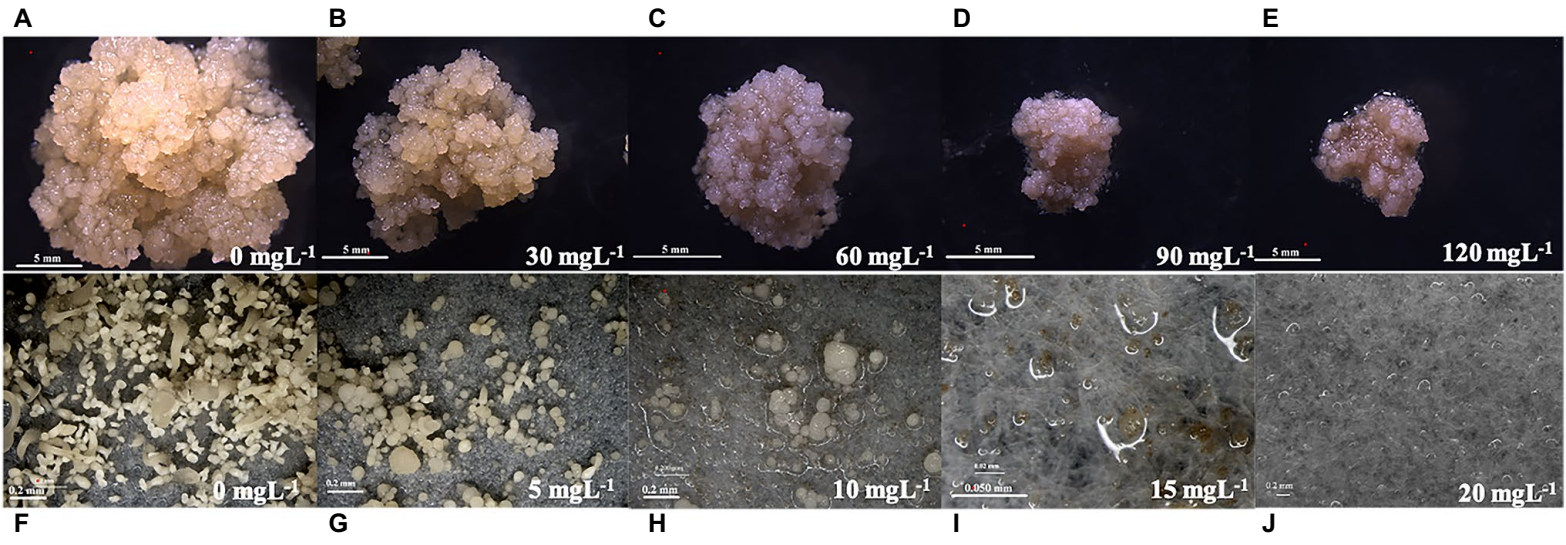
Effect of different mass concentrations of Geneticin (G418) on the growth of embryogenic calli and somatic embryo regeneration of the *Liriodendron* hybrid. Plate images showing the proliferation and tolerance level of the callus (**A–E**: 0, 30, 60, 90, and 120 mg L^−1^; bar: 5 mm), and somatic embryos [**F–J**: 0, 5, 10, 15, and 20 mgl^−1^; **(F–H,J)**: bar: 0.2 mm; I: bar: 0.05 mm] under selective conditions.

G418 sensitivity testing was carried out to determine the optimal concentration that arrested the growth of embryonic callus. With a culture duration of 1–8 weeks, as the G418 concentration increased, the embryonic callus growth rate gradually decreased (Figure 2A) and the browning degree increased, with significant differences detected and some calli even dying during the process (Figures 3A–E). The induction of calli was completely inhibited by 90 mg L^−1^ G418 (Figure 3D). After treatment with 90 mg L^−1^ G418, the fresh weight of the secondary callus clumps did not increase significantly on the CSM. Thus, a concentration of 90 mg L^−1^ G418 was selected to identify resistant calli during the first step of genetic transformation.

The embryogenic calli were incubated and proliferated on the EIM supplemented with different concentrations of G418, and the number of somatic embryos that were regenerated was estimated (Figure 2B). When the embryonic calli were cultured on the EIM, 267 somatic embryos were regenerated (Figures 2B, 3F). With 5 m gl^−1^ G418 on the EIM, 39 somatic embryos were obtained (Figures 2B, 3G). With the increase in the concentration of G418 (Figures 3F–J), there was a rapid and significative decline in the regeneration frequency of the somatic embryos at 10 mg L^−1^ of G418, under which only 3–4 somatic embryos were observed (Figures 2B, 3H). G418 at 15 mg L^−1^ completely prevented somatic embryo growth (only 0–1) when cultured on the EIM (Figures 2B, 3I). The somatic embryo regeneration became seriously restricted, and the somatic embryos became bleached with the extension of culture time. Therefore, the optimum concentration for selecting the transgenic somatic embryos was 15 mg L^−1^ G418.

#### Optimization of *Liriodendron* Hybrid Transformation

**Figure 4:**
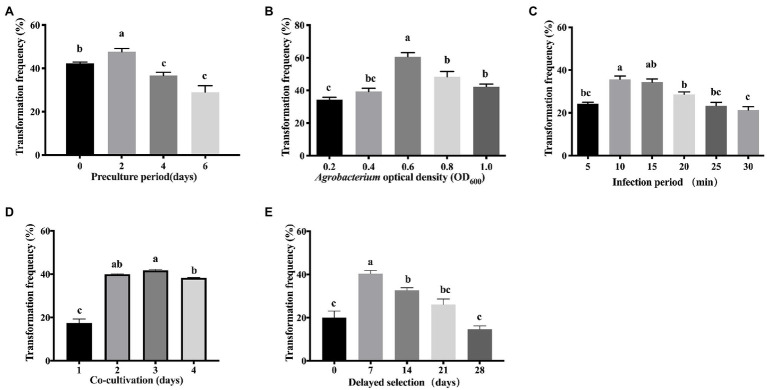
Effects of different factors in the transformation frequency of *Liriodendron* hybrid embryogenic callus. **(A)** Pre-culture period. **(B)** *Agrobacterium* optical density (OD_600_). **(C)** Infection period. **(D)** Co-cultivation period. **(E)** Delayed selection. Data correspond to the mean ± SD of three replicates. Column bars with the different letters indicate significant differences at p < 0.05 by ANOVA test.

To determine the influencing factors of pre-culture on the infection efficiency, the callus was incubated on pre-culture medium for 0, 2, 4, or 6 days, with no pre-cultured callus used as a control (Figure 4A). It was found that 2-day pre-culture resulted in a significantly (*p* < 0.05) higher transformation efficiency (47.7%) and more GUS-positive callus cells than the control (42.3%), and the 4-day (36.7%) and 6-day (29%) pre-culture. A longer pre-culture period for 4–6 days resulted in decreased transformation frequency. Therefore, 2 days of pre-culture at CCM was used for optimization (Figure 4A).

The density of *A. tumefaciens* significantly affected the transformation efficiency. The bacterial density of OD_600_ = 0.6 was significantly (p < 0.05) higher compared with OD_600_ values in the range of 0.2–0.4 and 0.8–1.0 (Figure 4B), and produced the most *GUS*-positive cells, at which the maximum transformation efficiency (60.7%) was obtained. The transformation efficiency of 34% was obtained when OD_600_ = 0.2, and 39% was obtained in Agrobacteria with OD_600_ = 0.4. The transformation efficiency was significantly lower than OD_600_ = 0.6, whereas the transformation efficiency was not significantly different between the higher concentrations of OD_600_ = 0.8 (48%) and 1.0 (42%). Therefore, OD_600_ = 0.6 was used for transforming embryogenic calli of the *Liriodendron* hybrid.

We tested the effect of infection duration on Agrobacterium transformation. When the infection duration was 10 min in the transformation experiment of the calli, the highest transformation frequency (35.7%) was recorded, but no significant difference in transformation efficiency was detected between the infection duration at 10 and 15 min, after which it then further decreased, which was unsuitable due to the uncontrollable overgrowth of the bacteria (Figure 4C). Prolonging the infection duration to 20 min resulted in reduced transformation efficiency (28%). The frequency of transformation was less than 23% when the infection duration was longer than 25 min. A longer infection period (30 min) significantly decreased (*p* < 0.05) the transformation efficiency due to cell necrosis.

After infection, the effect of the co-cultivation period on the efficiency of transformation was evaluated. The highest transformation frequency (43%) was obtained when the callus was co-cultivated on CCM for 3 days (Figure 4D), while shorter periods and prolonged co-cultivation resulted in reduced transformation efficiency (Figure 4D). Co-cultivation for 1 day resulted in significantly reduced efficiency of transient transformation at 17%, whereas co-cultivation for 2 days resulted in reduced transformation frequency (42.7%). When the co-cultivation duration was extended to 4 days, the transformation efficiency was reduced (39%), due to rapid bacterial overgrowth around the calli.

The delayed selection period is a critical factor in the genetic transformation of calli. Without delayed selection, the average transformation frequency was 20% (Figure 4E). Seven days of delayed selection improved the transgenic efficiency significantly (40.3%; Figure 4E). Increasing the length of the delayed selection period to 14–28 days significantly (*p* < 0.05) reduced the transformation frequency to 32.7% at 14 days, 26% at 21 days, and 14.7% at 28 days, while a significant difference was observed between 0 and 7 days of delayed selection (Figure 4E).

Thus, the best results in terms of the induction of a *GUS*-positive callus were obtained under 2 days of pre-culture on CCM, a bacterial density of OD_600_ = 0.6 for 10 min, co-cultivation of the explants in darkness for 3 days, and delaying selection for 7 days at 23°C. This approach produced the most *GUS*-positive cells and was used in subsequent optimization experiments.

#### Development of the Transgenic Plants

**Figure 5:**
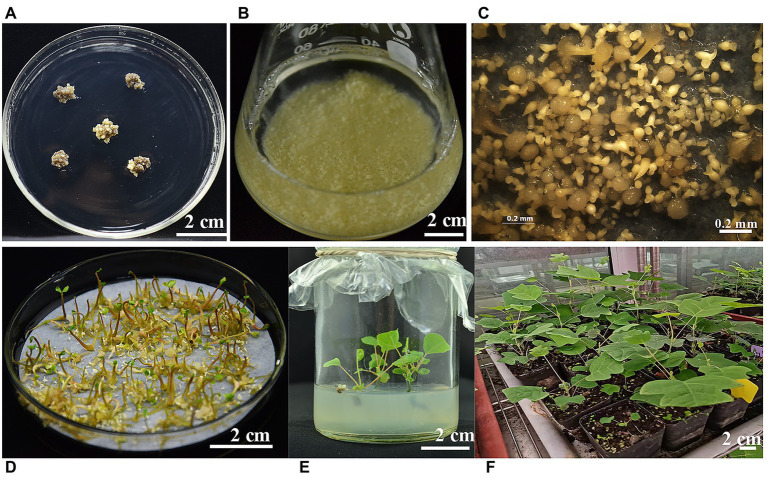
The different developmental stages of transgenic plant regeneration from calli of the *Liriodendron* hybrid. **(A)** Geneticin (G418)-resistant embryogenic callus was cultured on callus selection medium (CSM). **(B)** Suspension culture embryogenic calli in liquid CSM. **(C)** Cotyledonary stage embryos were induced on embryo induction medium (EIM). **(D)** G418-resistant embryo seedlings. **(E)** Regenerating embryo seedlings were cultured on shoot elongation medium (SEM). **(F)** Regenerating transgenic plantlets growing in the greenhouse. **(A,B,D,E,F)**: scale bar = 2 cm; **(C)**: scale bar = 0.2 mm.

After delaying selection for 7 days, the G418-resistant new callus began to emerge from the browning and necrotic callus that had been infected with *Agrobacterium*. After subculture for 8 weeks (Figure 5A), the G418-resistant calli were transferred to CSM. Through artificial dispersion, suspended calli in liquid medium were cultured on a rotary shaker at 23°C in the dark for 2 weeks (Figure 5B). Then, the suspension cells were collected and transferred to EIM with antibiotic. After culturing for 3–4 weeks in the dark, G418-resistant somatic embryos were regenerated from the embryogenic callus (Figure 5C). After light culture on EIM for 2–3 weeks, the cotyledons of the G418-resistant seedlings rapidly turned green (Figure 5D), whereas all presumed non-transgenic somatic embryos grew slowly or died due to the presence of antibiotics. After the seedlings had grown to a height of 3–5.0 cm in the medium (Figure 5E), the plantlets were transferred to soil pots in the greenhouse (Figure 5F).

#### Histochemical GUS Assay

**Figure 6:**
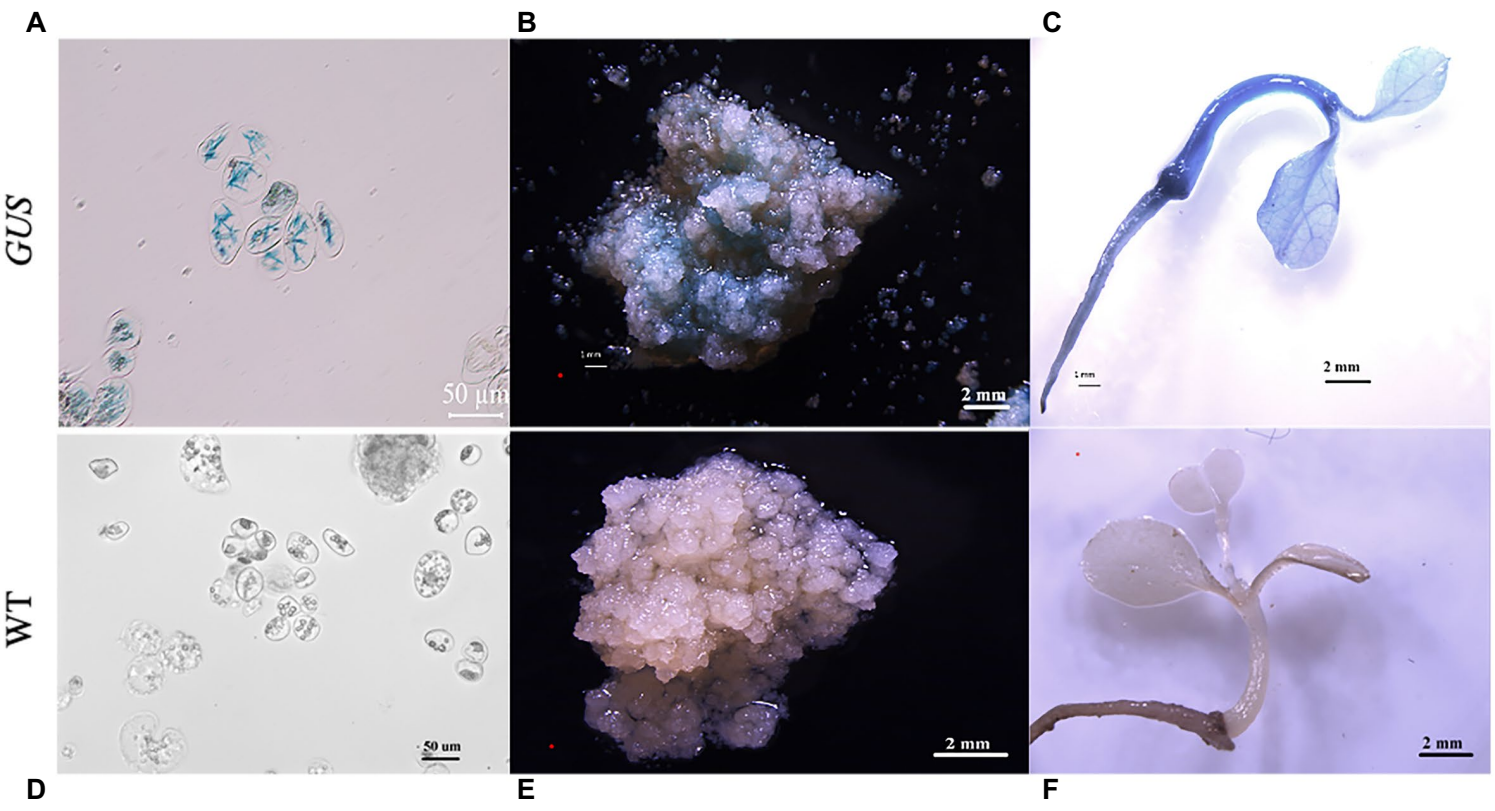
Histochemical staining for *ß*-glucuronidase (*GUS*) activity in the transgenic plants. Histochemical staining for *GUS* activity in transformed single cells **(A)**, callus clumps **(B)**, and plants **(C)**. **(D)** Histochemical staining for *GUS* activity in the non-transformed single cells (control). **(E)** Non-transformed callus clumps (control) and **(F)** plants. **(A,D)**: scale bar = 50 μm; **(B,C,E,F)** scale bar = 2 mm.

The transient *GUS* activity of the single suspension cells was determined in *A. tumefaciens*-inoculated calli immediately after co-cultivation with *Agrobacterium* (Figure 6A). Histochemical *GUS* assay showed that the callus clumps were mostly stained intensely blue (Figure 6B).

*GUS* staining indicated that *GUS* was expressed in the G418-resistant plantlets. Stable *GUS* activity was detected in the roots, stems, and leaves of all the selected G418-resistant plants (Figure 6C), whereas no *GUS* expression was observed in the non-transformed (control) single suspension cells, calli, and plantlets (Figures 6D–F).

#### Molecular Analysis of Transformants

**Figure 7:**
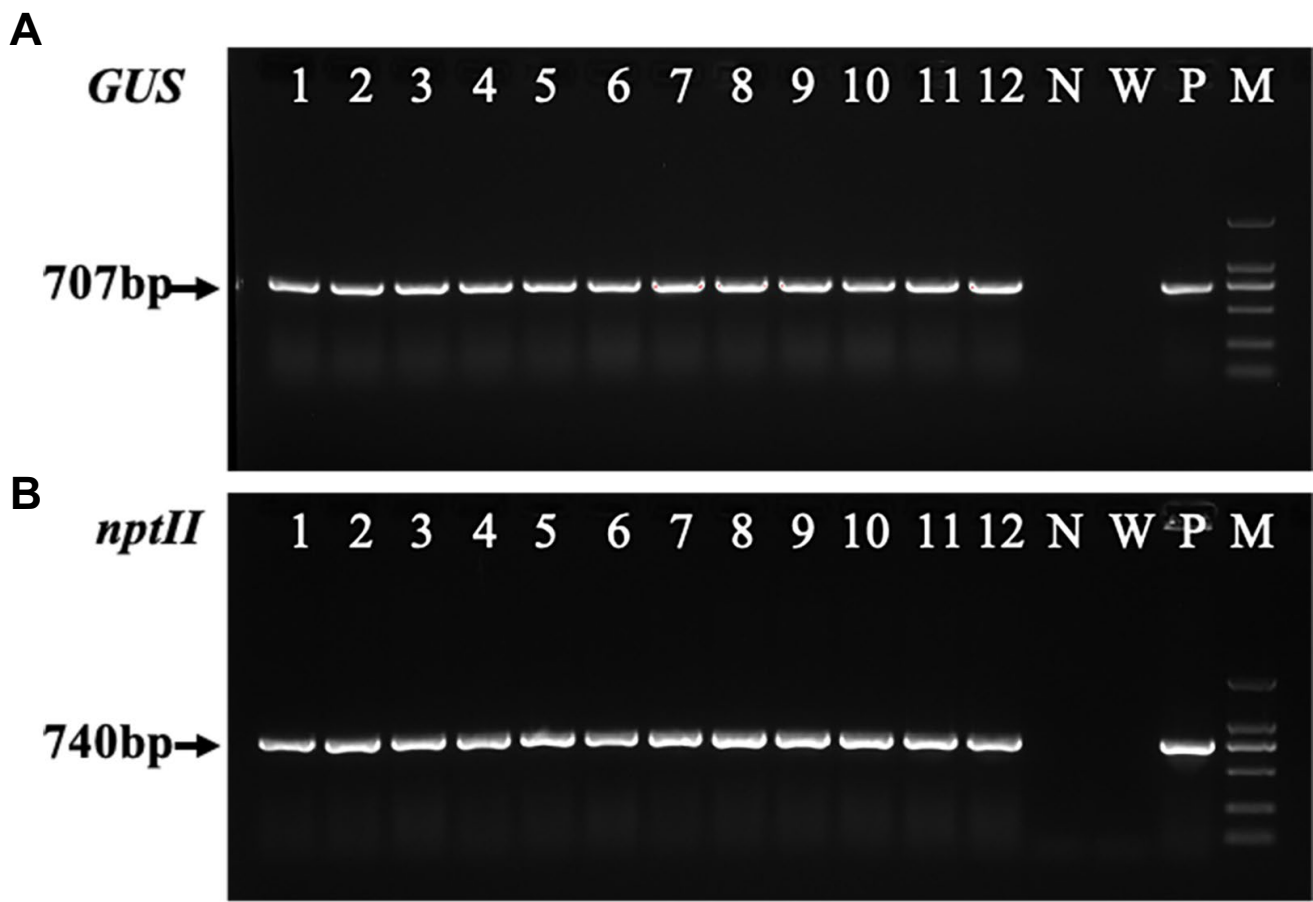
Polymerase chain reaction (PCR) analysis of the transgenic calli and transgenic plants. **(A)** The PCR analysis of the *ß*-glucuronidase (*GUS*) gene (707 bp) isolated from transgenic calli and the leaves of the transgenic lines and non-transformed plants of the *Liriodendron* hybrid. **(B)** PCR amplification of the *NPT-II* gene (740 bp) from the transgenic and non-transgenic lines. Lines 1–3 are transgenic calli, and lines 4–12 are transgenic plants. N, negative control (non-transgenic sample). W, water. P, positive control (plasmid). M, DL2000 marker.

The transformant was amplified using the vector primer pair to confirm that *GUS* and *NPT-II* were inserted into the *Liriodendron* hybrid genome. A 707-bp fragment of the expected size was detected in all transgenic lines (Figure 7A, lanes 1–12) and the positive control (Figure 7A, lane P), whereas no corresponding fragments were amplified in the non-transformed plant line and water samples (Figure 7A, lanes N, W). A single expected band of about 740 bp (Figure 7B) was amplified from the transgenic lines using the *NPT-II* primers to check the transgene in the transgenic plants. Through PCR analysis, PCR-positive transformant frequencies were generated (Figures 7A,B), and approximately 97.5% of the selected plants grew vigorously.

**Figure 8:**
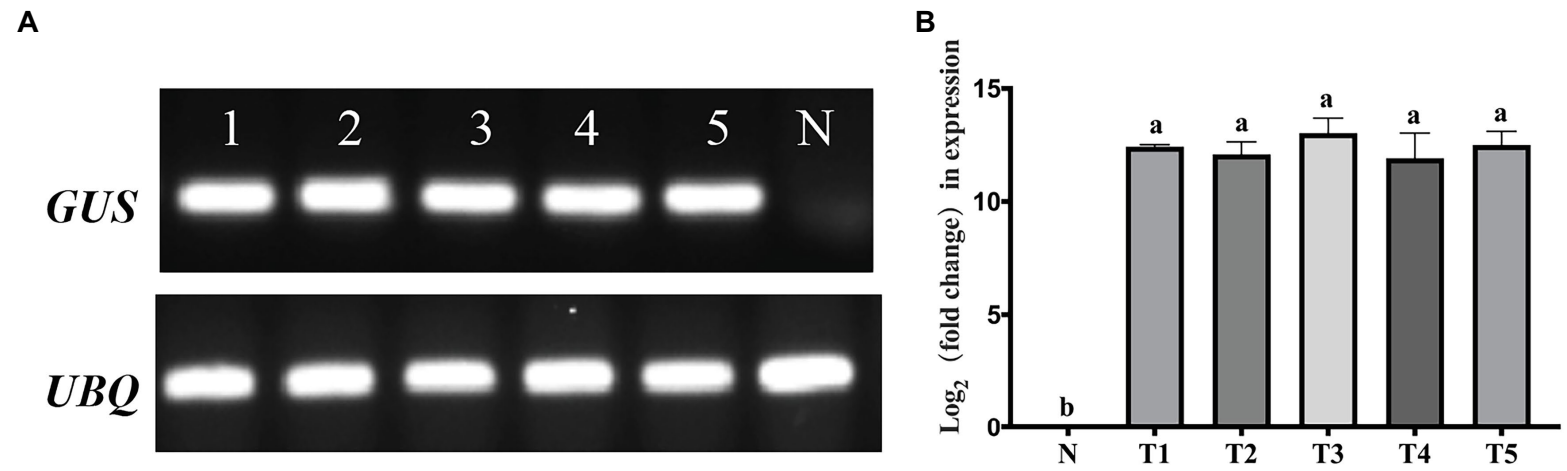
Polymerase chain reaction (PCR) detection of the *ß*-glucuronidase (*GUS*) gene at the RNA level of transgenic lines. **(A)** Real-time (RT)-PCR detection of the *GUS* gene. **(B)** Quantitative real-time (qRT)-PCR determined the levels of the *GUS* gene. N, negative control (non-transgenic sample). Lines 1–5 and T1–T5 represent transgenic plants. A one-way analysis of variance (ANOVA) test was used for statistical analysis. Values marked with different letters indicate statistical significance at *p <* 0.05 by Tukey test.

The RT–PCR (Figure 8A) and qRT–PCR (Figure 8B) analysis further confirmed the expression of the *GUS* gene in the five *GUS*-positive (PCR) transgenic plants, and the *GUS* gene was not detected in the non-transformed plants.

**Figure 9:**
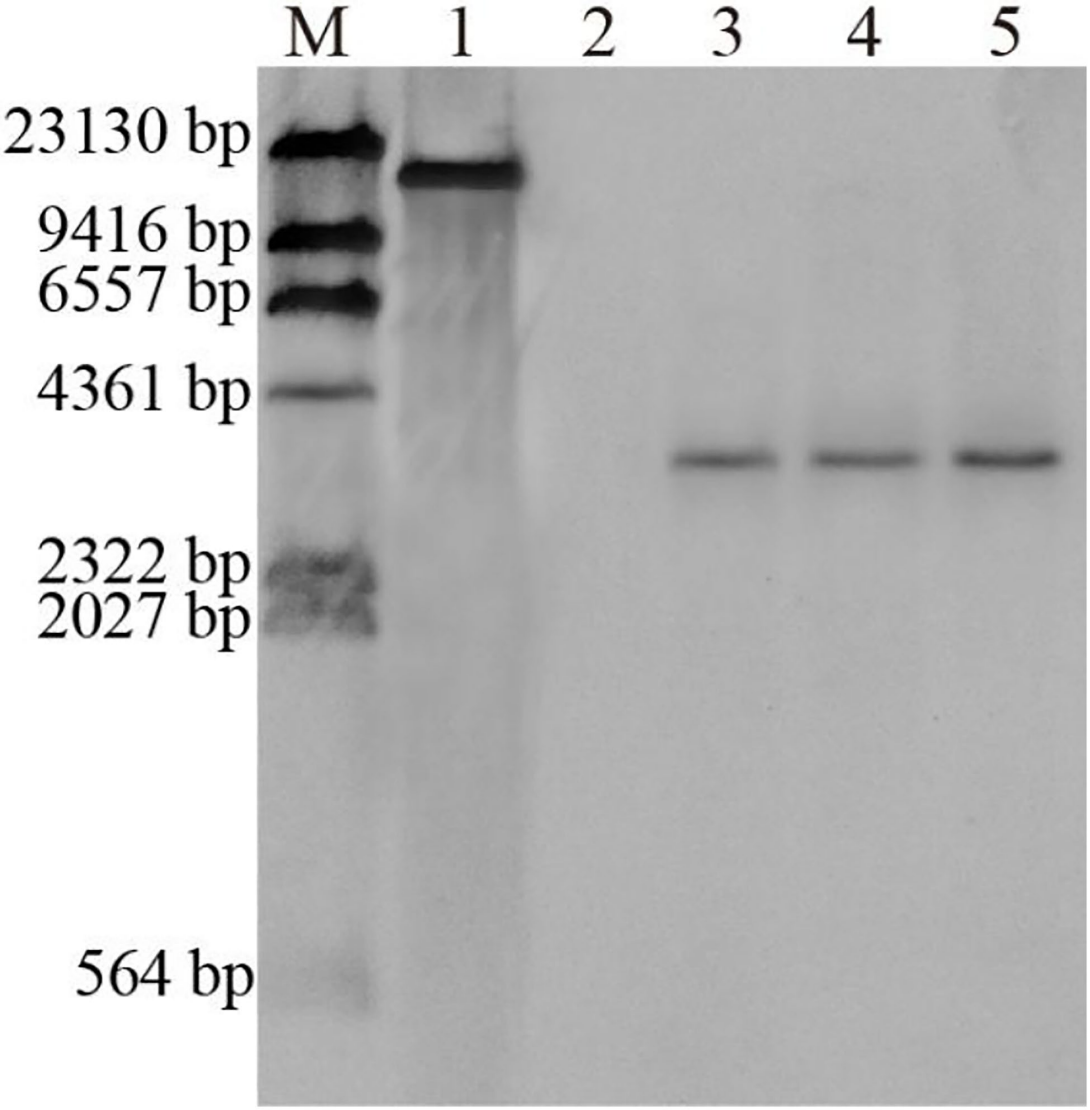
Southern hybridization of genomic DNA from non-transformed and transgenic plants of the *Liriodendron* hybrid. Lane M, DNA Molecular-Weight Marker, DIG-labeled; Lane 1, plasmid DNA (positive control); Lane 2, non-transformed plants; Lanes 3–5, transformed plants.

Southern blot analysis of the putative transgenic plants confirmed that the *GUS* gene was integrated into the transgenic plant genome (Figure 9). Genomic DNA was digested with HindIII (a single site), the number of bands revealed the copy number of the probe with the *GUS* gene coding region, and in transgenic lines of the *Liriodendron* hybrid, lane 3, lane 4 and lane 5 exhibited single copy insertion. No hybridization signal was detected for the non-transformed plants. Southern blot hybridization indicated that these plants represented transformation events.

These results demonstrated that the *GUS* gene had successfully integrated into the transgenic plant genome and was expressed.

## Discussion

### Transformation Efficiencies Depend on an Efficient *in vitro* Culture System

Following the genome sequencing of *Liriodendron* species, assessing the function of all the predicted genes has remained one of the main challenges, with the primary obstacle being the low frequency of transgenic plants. The transformation protocols rely on the availability of regenerative explant sources, and is a time-consuming process to produce transgenic individuals (Song et al., 2019). Embryogenic calli have been demonstrated to be the best target explants for *Agrobacterium*-mediated transformation in some plant species (Dutt and Grosser, 2010; Yang et al., 2010; Sun et al., 2020). Somatic embryogenesis is an ideal tool to be used in the study of genetic transformation. A transformation frequency of 25% was obtained with embryogenic material in European chestnut (Corredoira et al., 2004), while a transformation efficiency of 14.2% was achieved by somatic embryogenesis in *Morus alba* (Agarwal and Kanwar, 2007), and a transformation rate of up to 43% was obtained with embryogenic callus cultures as a target material in *Cyclamen persicum* (Ratjens et al., 2018). We previously established a high-frequency somatic embryogenesis system (Li et al., 2012), which was one of the critical factors influencing the success of plant genetic transformation in this study.

### High-Efficiency *Agrobacterium*-Mediated Transformation of the *Liriodendron* Hybrid

The high-efficiency genetic transformation of *Liriodendron* species requires the availability of efficient methods for gene transfer, plant regeneration, and transgenic plant selection. Based on this protocol, we improved the efficiency of transgenic transformation from various perspectives, including G418 concentration, pre-culture, cell density, co-culture conditions, and selection regime.

Research has indicated that explant pre-culture is a critical parameter in the *Agrobacterium*-mediated genetic transformation of several plant species (Barik et al., 2005; Xu et al., 2009; Kumar et al., 2010). In this study, the highest frequency of G418-resistant calli was observed when the embryogenic calli were pre-cultured for 2 days prior to infection with *A. tumefaciens*. These results indicated that pre-culture enabled the callus to sufficiently withstand bacterial infection and also enhanced the frequency of *GUS* expression.

The *Agrobacterium* cell density and infection time affect the frequency of transformation. However, higher *Agrobacterium* cell density and infection time and prolonged co-culture result in *Agrobacterium* strain overgrowth and embryogenic callus necrosis, thereby reducing the transformation frequency in plant species (Yang et al., 2010; Niedbała et al., 2021). Longer periods of co-culture seem to be helpful to achieve the transfer of T-DNA into plant cells. When the co-cultivation duration exceeded 3 days, the remaining *Agrobacterium* strains that attached onto the surface of the embryogenic calli overgrew and were not easily eliminated by 400 mg L^−1^ cefotaxime during subsequent selective culture, resulting in many calli becoming dark brown or black in color and ultimately dying. In the present study, we found that transferring the infected calli to CCM on filter paper was effective for decreasing bacterial growth (data not shown). An increase or decrease in the optical density of the *Agrobacterium* inoculum was not conducive to transformation. One reason for this finding may have been bacterial overgrowth, while extensive embryogenic callus necrosis occurred at optical density values greater than 0.6 (Saini and Jaiwal, 2007; Li et al., 2017).

### Selective Pressure Is Important for Successful Genetic Transformation

The selection of target plant materials is particularly important for successful genetic transformation (Wang et al., 2005). In our previous work, we found that the embryonic callus of the *Liriodendron* hybrid had strong kanamycin resistance, and a kanamycin concentration of 400 mg L^−1^ could not consistently inhibit callus growth (data not shown). The process of screening is long and the effects of screening are not always obvious. Therefore, in this study G418 was used as the selective agent in the genetic transformation method instead of kanamycin to explore the G418 sensitivity of the embryonic callus and somatic embryogenesis of *Liriodendron*. The result obtained from G418 sensitivity testing indicated that embryogenic callus proliferation was the least sensitive stage to G418, while somatic embryo regeneration was more sensitive. A previous study also demonstrated that different developmental stages of plant tissue culture revealed various tolerances to the same type of selective agent (Wu et al., 2021). Adjusting the selective agent concentration to balance the selection efficiency and transgenic plantlet regeneration is critical to obtaining positive transformed plantlets (Liu et al., 2021).

Therefore, this study developed a step-down concentration of the selective agent in the selective culture process. A novel three-stage transformant selection strategy was applied at different developmental stages due to the differences in G418-resistant embryonic callus propagation and somatic embryogenesis. G418 selection began 7 days after co-cultivation with *Agrobacterium*, and the transgenic efficiency improved significantly (Figure 4E). Initially, the co-cultivated embryogenic calli were transferred to DSM, and after culture for 7 days, the embryogenic calli were transferred onto CSM containing 90 mg L^−1^ G418 and 200 mg L^−1^ cefotaxime. Finally, the resistant embryogenic calli were suspension-cultured for 2 weeks, and then the suspension cells were cultured on EIM supplemented with 15 mg L^−1^ G418 and 200 mg L^−1^ cefotaxime to induce somatic embryos. The delayed selection strategy significantly improved the frequency of *Agrobacterium*-mediated transformation in the *Liriodendron* hybrid.

It is possible that delayed selection permits transformed callus division, resulting in the formation of multi-cell clusters that may be able to withstand selection pressure better than single cells (Yao et al., 1995). In addition, the selection duration was delayed to 21–28 days, and with the extension of the delayed selection duration, the regeneration rate increased. However, beyond a certain time point (21–28 days), many non-transformed embryogenic calli were induced. This result confirmed that developmental phases differed in their G418 sensitivity.

### Confirmation of Transgenic Plants

Several PCR-negative plants and the likelihood of regenerating chimeras were eliminated by a second round of selection pressure applied stringently in callus culture and somatic embryogenesis when the somatic embryos were induced on the EIM containing 15 mg L^−1^ G418. Selecting transgenic lines is important after transformation, and second-round selection can eliminate the PCR-negative transgenic plants. The RT-PCR and qRT-PCR analysis indicated that the *GUS* gene was expressed, and reported the levels of expression of the transgenes. Southern blot analysis was carried out to confirm the *GUS* gene integration, and indicated that transgenic events occurred. Together with the expression of the *GUS* gene, which was confirmed by histochemical *GUS* assays, the results demonstrated that the *GUS* gene was integrated into the *Liriodendron* hybrid genome.

## Conclusion

The breeding of almost all woody plants is limited by high heterozygosity, a long juvenile period, and self-incompatibility (Tang et al., 2007). A transgenic regeneration system is a promising tool for modifying tree species to increase resistance to biotic and abiotic stresses and enhance productivity, and also provides an opportunity to eliminate the limitations of traditional breeding and accelerate germplasm improvement (Pérez-Clemente et al., 2005).

In this study, a reliable and stable *A. tumefaciens*-mediated transformation system was established in the *Liriodendron* hybrid using embryogenic calli. The overall duration of the genetic transformation process and plant regeneration through the somatic embryogenesis process was very short (5 months). Transformed calli could still regenerate transgenic somatic embryos after 3 years of culture under G418 selection (data not shown). This method would allow researchers to obtain enough embryogenic material through embryogenic callus suspension culture for future genetic engineering improvements within a short time. Moreover, the proposed protocol enables target genes to be transferred into *Liriodendron* species and may be applicable for the transformation of other tree species.

In conclusion, we have reported an efficient short-term transgenic regeneration system for genes introduced into the *Liriodendron* hybrid mediated by optimizing different parameters considered crucial for transformation. Delayed selection was found to be the most important parameter affecting the efficiency of transformation.

## Data Availability Statement

The original contributions presented in the study are included in the article/**Supplementary Material**, further inquiries can be directed to the corresponding author.

## Author Contributions

JC and JS contributed the conception and design of the study. ML, DW, XL, YL, YZ, YP, and TC performed the experiments. ZH carried out the statistical analysis. ML wrote the manuscript. All authors contributed to the article and approved the submitted version.

## Funding

This work was supported by the National Key R&D Program of China (2021YFD2200102), the Nature Science Foundation of China (32071784), the Priority Academic Program Development of Jiangsu Higher Education Institutions (PAPD), and Natural Science Foundation of Jiangsu Province (Grant No. BK20180313).

## Conflict of Interest

The authors declare that the research was conducted in the absence of any commercial or financial relationships that could be construed as a potential conflict of interest.

## Publisher’s Note

All claims expressed in this article are solely those of the authors and do not necessarily represent those of their affiliated organizations, or those of the publisher, the editors and the reviewers. Any product that may be evaluated in this article, or claim that may be made by its manufacturer, is not guaranteed or endorsed by the publisher.

## References

[ref1] Agarwal S. Kanwar K. (2007). Comparison of genetic transformation in *Morus alba* L. via different regeneration systems. Plant Cell Rep. 26, 177–185. doi: 10.1007/s00299-006-0217-3, PMID: 16951950

[ref2] Barik D. P. Mohapatra U. Chand P. K. (2005). Transgenic *grasspea* (*Lathyrus sativus* L.): factors influencing *agrobacterium*-mediated transformation and regeneration. Plant Cell Rep. 24, 523–531. doi: 10.1007/s00299-005-0957-5, PMID: 15948005

[ref3] Bett B. Gollasch S. Moore A. Harding R. Higgins T. J. V. (2019). An improved transformation system for cowpea (*Vigna unguiculata* L. Walp) via sonication and a kanamycin-Geneticin selection regime. Front. Plant Sci. 10:219. doi: 10.3389/fpls.2019.00219, PMID: 30873198 PMC6401653

[ref4] Bolyard M. G. Hajela R. K. Sticklen M. B. (1991). Microprojectile and *Agrobacterium*-mediated transformation of pioneer elm. J. Arboric. 31, 1313–1317. doi: 10.1007/s11738-009-0365-5

[ref5] Cha T. S. Yee W. Aziz A. (2012). Assessment of factors affecting *agrobacterium*-mediated genetic transformation of the unicellular green alga, *Chlorella vulgaris*. World J. Microbiol. Biotechnol. 28, 1771–1779. doi: 10.1007/s11274-011-0991-0, PMID: 22805959

[ref6] Chen Z. Chen J. Li T. Wu C. Jisen S. (2007a). Study on factors influencing transformation of *Liriodendron* hybrids (*L. chinense × L. tulipifera*) with bivalent disease resistant genes. Mol. Plant Breed. 5, 588–592. doi: 10.3969/j.issn.1672-416X.2007.04.024

[ref7] Chen J. Hao Z. Guang X. Zhao C. Wang P. Xue L. . (2019a). *Liriodendron* genome sheds light on angiosperm phylogeny and species–pair differentiation. Nat. Plants 5, 18–25. doi: 10.1038/s41477-018-0323-6, PMID: 30559417 PMC6784881

[ref8] Chen Z. Jinhui C. Jisen B. L. L. T. W. C. S. (2007b). Establishment of embryogenic callus suspension culture system in *Liriodendron* hybrids. Mol. Plant Breed. 5, 137–140. doi: 10.3969/j.issn.1672-416X.2007.01.025

[ref9] Chen J. Shi J. Zhuge Q. Huang M. (2003a). Studies on the somatic embryogenesis of *Liriodendron* hybrids (*L. chinense × L. tulipifera*). Sci. Silvae Sin. 15, 282–285. doi: 10.1007/BF02974893

[ref10] Chen P.-Y. Wang C.-K. Soong S.-C. To K.-Y. (2003b). Complete sequence of the binary vector pBI121 and its application in cloning T-DNA insertion from transgenic plants. Mol. Breed. 11, 287–293. doi: 10.1023/A:1023475710642

[ref11] Chen T. Wang P. Zhang J. Shi J. Cheng T. Chen J. (2019b). Effects of combined ABA and ZT treatment on somatic embryogenesis and development of *Liriodendron sino-americanum*. Sci. Silvae Sin. 55, 64–71. doi: 10.11707/j.1001-7488.20190307

[ref12] Cheng M. Hu T. Layton J. Liu C.-N. Fry J. E. (2003). Desiccation of plant tissues post-*agrobacterium* infection enhances T-DNA delivery and increases stable transformation efficiency in wheat. In Vitro Cell Dev. Biol. Plant 39, 595–604. doi: 10.1079/ivp2003471

[ref13] Corredoira E. Montenegro D. San-José M. Vieitez A. M. Ballester A. (2004). *Agrobacterium*-mediated transformation of European chestnut embryogenic cultures. Plant Cell Rep. 23, 311–318. doi: 10.1007/s00299-004-0804-0, PMID: 15338188

[ref14] Dai J. Vendrame W. A. Merkle S. A. (2004). Enhancing the productivity of hybrid yellow-poplar and hybrid sweetgum embryogenic cultures. In Vitro Cell Dev. Biol. Plant 40, 376–383. doi: 10.1079/ivp2004538

[ref15] Dutt M. Grosser J. W. (2010). An embryogenic suspension cell culture system for *Agrobacterium*-mediated transformation of citrus. Plant Cell Rep. 29, 1251–1260. doi: 10.1007/s00299-010-0910-0, PMID: 20711728

[ref16] Fang Yuan Bingying Leng Haonan Zhang Wang Guoliang, Han, and Baoshan (2019). A WD40-repeat protein From the Recretohalophyte *Limonium* bicolor enhances trichome formation and salt tolerance in *Arabidopsis*. Front. Plant Sci. 10, 1456–1456. doi:10.3389/fpls.2019.01456, PMID: 31781150 PMC6861380

[ref17] Fillatti J. A. J. Sellmer J. Mccown B. Haissig B. Comai L. (1987). Agrobacterium mediated transformation and regeneration of populus. Mol. Gen. Genet. 206, 192–199. doi: 10.1007/BF00333574

[ref18] Hao Z. Liu S. Hu L. Shi J. Chen J. (2020). Transcriptome analysis and metabolic profiling reveal the key role of carotenoids in the petal coloration of *Liriodendron tulipifera*. Hortic Res. 7:70. doi: 10.1038/s41438-020-0287-3, PMID: 32377360 PMC7193617

[ref19] Hooykaas P. J. J. Klapwijk P. M. Nuti M. P. Schilperoort R. A. Rorsch A. (1977). Transfer of the *Agrobacterium tumefaciens* TI plasmid to avirulent agrobacteria and to rhizobium *ex planta*. J. Gen. Microbiol. 98, 477–484. doi: 10.1099/00221287-98-2-477

[ref20] Huo A. Chen Z. Wang P. Yang L. Wang G. Wang D. . (2017). Establishment of transient gene expression systems in protoplasts from *Liriodendron* hybrid mesophyll cells. PLoS One 12:e0172475. doi: 10.1371/journal.pone.0172475, PMID: 28323890 PMC5360215

[ref21] Jefferson R. A. (1987a). Assaying chimeric genes in plants: The *GUS* gene fusion system. Plant Mol. Biol. Report. 5, 387–405. doi: 10.1007/BF02667740

[ref22] Jefferson R. A. (1987b). GUS fusions: beta-glucuronidase as a sensitive and versatile gene fusion marker in higher plants. EMBO J. 6, 3901–3907. doi: 10.1089/dna.1987.6.583, PMID: 3327686 PMC553867

[ref23] Karami O. (2008). Factors affecting *agrobacterium*-mediated transformation of plants. Transgenic Plant J. 2, 127–137.

[ref24] Kumar N. Vijay Anand K. G. Pamidimarri D. V. N. S. Sarkar T. Reddy M. P. Radhakrishnan T. . (2010). Stable genetic transformation of *Jatropha curcas* via *Agrobacterium tumefaciens*-mediated gene transfer using leaf explants. Ind. Crop. Prod. 32, 41–47. doi: 10.1016/j.indcrop.2010.03.002

[ref25] Li T. Chen J. Qiu S. Zhang Y. Wang P. Yang L. . (2012). Deep sequencing and microarray hybridization identify conserved and species-specific microRNAs during somatic embryogenesis in hybrid yellow poplar. PLoS One 7:e43451. doi: 10.1371/journal.pone.0043451, PMID: 22952685 PMC3430688

[ref26] Li S. Cong Y. Liu Y. Wang T. Shuai Q. Chen N. . (2017). Optimization of *Agrobacterium*-mediated transformation in soybean. Front. Plant Sci. 8:246. doi: 10.3389/fpls.2017.00246, PMID: 28286512 PMC5323423

[ref27] Li T. Jisen S. Jinhui C. Limin B. Zhi C. Chun W. (2007). Developmental synchronization of somatic embryogenesis under suspension culture condition. Mol. Plant Breed. 5, 436–442. doi: 10.3969/j.issn.1672-416X.2007.03.024

[ref28] Liu G. Yuan Y. Jiang H. Bao Y. Ning G. Zhao L. . (2021). *Agrobacterium tumefaciens*-mediated transformation of modern rose (*Rosa hybrida*) using leaf-derived embryogenic callus. Hortic Plant J. 7, 359–366. doi: 10.1016/j.hpj.2021.02.001

[ref29] Matsunaga E. Nanto K. Oishi M. Ebinuma H. Morishita Y. Sakurai N. . (2012). *Agrobacterium*-mediated transformation of *Eucalyptus globulus* using explants with shoot apex with introduction of bacterial choline oxidase gene to enhance salt tolerance. Plant Cell Rep. 31, 225–235. doi: 10.1007/s00299-011-1159-y, PMID: 22009051

[ref30] Merkle S. A. Hoey M. T. Watson-Pauley B. A. Schlarbaum S. E. (1993). Propagation of *Lirlodendron* hybrids via somatic embryogenesis. Plant Cell Tissue Organ Cult. 34, 191–198. doi: 10.1007/BF00036101

[ref31] Merkle S. A. Sommer H. E. (1986). Somatic embryogenesis in tissue cultures of *Liriodendron tulipifera*. Can. J. For. Res. 16, 420–422. doi: 10.1139/x86-077

[ref32] Murashige T. Skoog F. (1962). A revised medium for rapid growth and bio assays with tobacco tissue cultures. Physiol. Plant. 15, 473–497. doi: 10.1111/j.1399-3054.1962.tb08052.x

[ref33] Niedbała G. Niazian M. Sabbatini P. (2021). Modeling *agrobacterium*-mediated gene transformation of tobacco (*Nicotiana tabacum*)—a model plant for gene transformation studies. Front. Plant Sci. 12:695110. doi: 10.3389/fpls.2021.695110, PMID: 34413865 PMC8370025

[ref34] Pamidimarri S. Boricha G. Muppala P. (2009). A simplified method for extraction of high quality genomic DNA from *Jatropha curcas* for genetic diversity and molecular marker studies. Indian J. Biotechnol. 28, 157–192. doi: 10.1089/hyb.2008.0100.MAb

[ref35] Pérez-Clemente R. M. Pérez-Sanjuán A. García-Férriz L. Beltrán J.-P. Cañas L. A. (2005). Transgenic peach plants (*Prunus persica* L.) produced by genetic transformation of embryo sections using the green fluorescent protein (GFP) as an *in vivo* marker. Mol. Breed. 14, 419–427. doi: 10.1007/s11032-005-0506-5

[ref36] Prasad B. Vadakedath N. Jeong H.-J. General T. Cho M.-G. Lein W. (2014). *Agrobacterium tumefaciens*-mediated genetic transformation of haptophytes (*Isochrysis species*). Appl. Microbiol. Biotechnol. 98, 8629–8639. doi: 10.1007/s00253-014-5900-7, PMID: 24993358

[ref37] Ratjens S. Mortensen S. Kumpf A. Bartsch M. Winkelmann T. (2018). Embryogenic callus as target for efficient transformation of *Cyclamen persicum* enabling gene function studies. Front. Plant Sci. 9:1035. doi: 10.3389/fpls.2018.01035, PMID: 30087683 PMC6066641

[ref38] Rugh C. Senecoff J. Meagher R. Merkle S. (1998). Development of transgenic yellow poplar for mercury phytoremediation. Nat. Biotechnol. 16, 925–928. doi: 10.1038/nbt1098-925, PMID: 9788347

[ref39] Saini R. Jaiwal P. K. (2007). *Agrobacterium tumefaciens*-mediated transformation of blackgram: an assessment of factors influencing the efficiency of *uidA* gene transfer. Biol. Plant. 51, 69–74. doi: 10.1007/s10535-007-0014-z 30754758

[ref40] Shin D.-I. Podila G. K. Huang Y. Karnosky D. F. (1994). Transgenic larch expressing genes for herbicide and insect resistance. Can. J. For. Res. 24, 2059–2067. doi: 10.1139/x94-264

[ref41] Shou H. Frame B. R. Whitham S. A. Wang K. (2004). Assessment of transgenic maize events produced by particle bombardment or *Agrobacterium*-mediated transformation. Mol. Breed. 13, 201–208. doi: 10.1023/B:MOLB.0000018767.64586.53

[ref42] Shrawat A. K. Becker D. Lörz H. (2007). *Agrobacterium tumefaciens*-mediated genetic transformation of barley (*Hordeum vulgare* L.). Plant Sci. 172, 281–290. doi: 10.1016/j.plantsci.2006.09.005 17177801

[ref43] Song G.-Q. Prieto H. Orbovic V. (2019). *Agrobacterium*-mediated transformation of tree fruit crops: methods, progress, and challenges. Front. Plant Sci. 10:226. doi: 10.3389/fpls.2019.00226, PMID: 30881368 PMC6405644

[ref44] Sun Z. L. Li X. Zhou W. Yan J. D. Gao Y. R. Li X. W. . (2020). *Agrobacterium*-mediated genetic transformation of Chinese chestnut (*Castanea mollissima* Blume). Plant Cell Tissue Organ Cult. 140, 95–103. doi: 10.1007/s11240-019-01713-4

[ref45] Tang W. Newton R. J. Weidner D. A. (2007). Genetic transformation and gene silencing mediated by multiple copies of a transgene in eastern white pine. J. Exp. Bot. 58, 545–554. doi: 10.1093/jxb/erl228, PMID: 17158108

[ref46] Thiruvengadam M. Jeyakumar J. Kamaraj M. Chung I. M. Kim J. J. (2013). Optimization of *agrobacterium*-mediated genetic transformation in gherkin (*Cucumis anguria* L.). Plant Omics 198, 765–776. doi: 10.1111/nph.12179

[ref47] Vidal N. Mallón R. Valladares S. Meijomín A. Vieitez A. (2010). Regeneration of transgenic plants by *agrobacterium*-mediated transformation of somatic embryos of juvenile and mature *Quercus robur*. Plant Cell Rep. 29, 1411–1422. doi: 10.1007/s00299-010-0931-8, PMID: 20972795

[ref48] Wang Z. (2003). The review and outlook on hybridization in tulip tree breeding in China. J. Nanjing Forest. Univ. 15, 282–285. doi: 10.1007/BF02974893

[ref49] Wang Q. Li P. Hanania U. Sahar N. Mawassi M. Gafny R. . (2005). Improvement of *agrobacterium*-mediated transformation efficiency and transgenic plant regeneration of *Vitis vinifera* L. by optimizing selection regimes and utilizing cryopreserved cell suspensions. Plant Sci. 168, 565–571. doi: 10.1016/j.plantsci.2004.09.033

[ref50] Wei Q. C. Chang L. P. (2009). Study on preparation and preservation method of competent cell of *Agrobacterium tumefaciens*. J. Anhui Agric. Sci. 37, 6342–6343. doi: 10.3969/j.issn.0517-6611.2009.14.016

[ref51] Wilde H.D. Meagher R.B. Merkle S.A. (1991). Transfer of Foreign Genes into Yellow-Poplar (*Liriodendron Tulipifera*). Springer, New York, NY.

[ref52] Wilde H. D. Meagher R. B. Merkle S. A. (1992). Expression of foreign genes in transgenic yellow-poplar plants. Plant Physiol. 98, 114–120. doi: 10.1104/pp.98.1.114, PMID: 16668600 PMC1080157

[ref53] Wu Y. Zhou N. Ni X. Okoye C. O. Jiang J. (2021). Developing a long-term and powerful in vitro culture and *agrobacterium*-mediated transformation system for *Miscanthus sinensis* (Poaceae). Ind. Crop. Prod. 161:113190. doi: 10.1016/j.indcrop.2020.113190

[ref54] Xia P. Hu W. Liang T. Yang D. Liang Z. (2020). An attempt to establish an *Agrobacterium*-mediated transient expression system in medicinal plants. Protoplasma 257, 1497–1505. doi: 10.1007/s00709-020-01524-x, PMID: 32564134

[ref55] Xu J. Wang Y. Z. Xia Yin H. Jing Liu X. (2009). Efficient *Agrobacterium tumefaciens*-mediated transformation of *Malus zumi* (Matsumura) Rehd using leaf explant regeneration system. Electron. J. Biotechnol. 12, 288–289. doi: 10.4067/S0717-34582009000100003

[ref56] Yang Y. Bao M. Liu G. (2010). Factors affecting *Agrobacterium*-mediated genetic transformation of embryogenic callus of *Parthenocissus tricuspidata* planch. Plant Cell Tissue Organ Cult. 102, 373–380. doi: 10.1007/s11240-010-9742-4

[ref57] Yang D. T. Lin S. S. Chen J. H. Yuan S. T. Shi J. S. Wang J. S. . (2015). (+)- and (−)-liriodenol, a pair of novel enantiomeric lignans from *Liriodendron* hybrid. Bioorg. Med. Chem. Lett. 25, 1976–1978. doi: 10.1016/j.bmcl.2015.03.015, PMID: 25817591

[ref58] Yao J. L. Cohen D. Atkinson R. Richardson K. Morris B. (1995). Regeneration of transgenic plants from the commercial apple cultivar Royal Gala. Plant Cell Rep. 14, 407–412. doi: 10.1007/BF00234044, PMID: 24185446

[ref59] Zhou Q. Dai L. M. Cheng S. Y. He J. Wang D. Zhang J. X. . (2014). A circulatory system useful both for long-term somatic embryogenesis and genetic transformation in *Vitis vinifera* L. cv. Thompson seedless. Plant Cell Tissue Organ Cult. 118, 157–168. doi: 10.1007/s11240-014-0471-y

[ref60] Zhou Y. Li M. Zhao F. Zha H. Yang L. Lu Y. . (2016). Floral nectary morphology and proteomic analysis of nectar of *Liriodendron tulipifera* Linn. Front. Plant Sci. 7:826. doi: 10.3389/fpls.2016.00826, PMID: 27379122 PMC4905952

