# Supplementary material for: Agrobacterium-Mediated Genetic Transformation of Embryogenic Callus in a Liriodendron Hybrid (L. Chinense × L. Tulipifera)
Source: Front Plant Sci. 2022 Mar 17;13:802128. doi: 10.3389/fpls.2022.802128 (PMC8970691; doi:10.3389/fpls.2022.802128)
Supplement: Supplementary file 1 [file Table_1.DOCX]

**Supplementary Table 1. Components of Murashige and Skoog (MS) medium**

| **Chemical** | **Manufacturer** | | **Quantities (per L)** | |
| --- | --- | --- | --- | --- |
| 20×MS Macro | Sinopharm Chemical Reagent Co., Ltd.，Shanghai, China |  | |  |
| NH_4_NO_3_ |  | 33g | |  |
| KNO_3_ |  | 38g | |  |
| MgSO_4_·7H_2_O |  | 7.4g | |  |
| KH_2_PO_4_ |  | 3.4g | |  |
| CaCl_2_ |  | 6.64g | |  |
| 100×MS Micro |  |  | |  |
| H_3_BO_3_ |  | 0.62g | |  |
| KI |  | 0.083g | |  |
| MnSO_4_·H_2_O |  | 1.69g | |  |
| ZnSO_4_·7H_2_O |  | 0.86g | |  |
| Na_2_MoO_4_ ·2H_2_O |  | 1mL (25 mg/mL stock) | |  |
| CuSO_4_·5H_2_O |  | 1mL (2.5 mg/mL stock) | |  |
| CoCl_2_·6H_2_O |  | 1mL (2.5 mg/mL stock) | |  |
| 100×MS Iron |  |  | |  |
| Na_2_EDTA·2H_2_O |  | 3.73g | |  |
| FeSO_4_·7H_2_O |  | 2.78g | |  |
| 100×MS Organic Constituents |  |  | |  |
| Thiamine hydrochloride (VB1) |  | 0.01g | |  |
| Nicotinic acid |  | 0.05g | |  |
| Pyridoxine hydrochloride (VB6) |  | 0.05g | |  |
| Glycine |  | 0.2g | |  |
| Inositol |  | 10g | |  |

Sinopharm Chemical Reagent Co., Ltd.is the producer company for the listed reagents.
