# Supplementary material for: Agrobacterium-Mediated Genetic Transformation of Embryogenic Callus in a Liriodendron Hybrid (L. Chinense × L. Tulipifera)
Source: Front Plant Sci. 2022 Mar 17;13:802128. doi: 10.3389/fpls.2022.802128 (PMC8970691; doi:10.3389/fpls.2022.802128)
Supplement: Supplementary file 2 [file Table_2.DOCX]

**Supplementary Table 2.** **Compositions of medium used in this study**

| **Chemical** | **Manufacturer** | **Quantities (per L)** | | | | | | | |
| --- | --- | --- | --- | --- | --- | --- | --- | --- | --- |
|  |  | | **CIM** | | **CCM** | **CSM** | **EIM** | **SEM** | **DSM** |
| 20×MS Macro |  | | | 50 mL | 50 mL | 50 mL | 50mL | 50mL | 50 mL |
| 100×MS Micro |  | | | 10 mL | 10 mL | 10 mL | 10mL | 10mL | 10 mL |
| 100×MS Iron |  | | | 10 mL | 10 mL | 10 mL | 10mL | 10mL | 10 mL |
| 100×MS Organic Constituents |  | | | 10 mL | 10 mL | 10 mL | 10mL | 10mL | 10 mL |
| Sucrose | Sinopharm Chemical Reagent Co., Ltd.，Shanghai,China | | | 30g | 30g | 30g | 30g | 30g | 30g |
| gelrite | Sigma-Aldrich, St. Louis, MO, United States | | | 2.3g | 2.3g | 2.3g | 2.3g | 2.3g | 2.3g |
| Vitamin C  (V_C_) | Sinopharm Chemical Reagent Co., Ltd.，Shanghai,China | | | 5 mg | 5 mg | 5 mg | 5 mg | 5 mg | 5 mg |
| 6-Benzyl-aminopurine (BA) | Sigma-Aldrich, St. Louis, MO, United States | | | 0.1mg | - | 0.1mg | - | - | 0.1mg |
| (2,4-Dichlor-phenoxy)-essigsaeure  （2,4-D） | Sigma-Aldrich, St. Louis, MO, United States | | | 0.5mg | - | 0.5mg | - | - | 0.5mg |
| N-Z-Amine A  （CH） | Sigma-Aldrich, St. Louis, MO, United States | | | 0.2g | - | 0.2g | 0.2g | - | 0.2g |
| (±)-cis,trans-Abscisic acid (ABA) | Sigma-Aldrich, St. Louis, MO, United States | | | - | - | - | 1mg | - | - |
| Acetosyringone (AS) | Sigma-Aldrich, St. Louis, MO, United States | | | - | 0.1mM | - | - | - | - |
| Cefotaxime sodium salt (Cef) | Sangon Biotech (Shanghai) Co., Ltd. Shanghai, China | | | - | - | 200mg | - | - | 400mg |
| Geneticin  (G418) | AMRESCO, LLC Solon, Ohio USA | | | - | - | 90 mg | - | - | - |

Note: All mediums, pH = 5.7 (adjusting by KOH)
